# Penile Fracture in a 55-Year-Old Man: A Report of a Rare Case

**DOI:** 10.7759/cureus.87730

**Published:** 2025-07-11

**Authors:** Abdul Safi, Jasmeet S Khera, Nik Furtado, Brian Peters

**Affiliations:** 1 Medical School, Creighton University School of Medicine, Omaha, USA; 2 Sports Medicine, Creighton University School of Medicine, Omaha, USA; 3 Internal Medicine, Creighton University School of Medicine, Omaha, USA; 4 Radiology, Creighton University School of Medicine, Omaha, USA

**Keywords:** corpus cavernosum rupture, penile fracture, penile injury, traumatic pelvic injury, tunica albuginea

## Abstract

Penile fracture is a rare urological emergency defined by the rupture of the tunica albuginea of an erect penis, most often due to blunt trauma during sexual intercourse. Prompt diagnosis and surgical repair are critical to prevent long-term complications such as erectile dysfunction and penile curvature. We present a rare case of penile fracture in a 55-year-old male with type 2 diabetes mellitus and a history of myocardial infarction, highlighting the diagnostic workup with ultrasound, MRI, surgical management, and recovery.

## Introduction

Penile fracture (PF) in an uncommon urological emergency that occurs when the tunica albuginea of an erect penis acutely ruptures due to trauma [1]. The classic mechanism is a bending injury from vigorous sexual intercourse, often with the partner on top, or other blunt trauma to an erect penis [2]. Clinically, patients typically report a sudden audible “crack” or popping sound, severe penile pain, and immediate detumescence, followed by rapid swelling and bruising of the penis. Between 9-20% of PF also present with urethral injury [3]. The incidence of PF has been estimated to be approximately 1.02 cases per 100,000 male population per year [4]. 

Advances in imaging now complement clinical diagnosis in ambiguous cases. While history and physical examination are diagnostic, adjunctive imaging can be done when the presentation is atypical or the patient’s examination is equivocal [5]. Ultrasound (US) can identify a tunica albuginea tear as a discontinuity or hematoma in the tunica outline [6]. Magnetic resonance imaging (MRI) offers superior soft-tissue contrast and can precisely delineate the site and extent of injury [2]. Current guidelines suggest using US as the initial imaging modality for suspected PF, with MRI reserved for cases where the diagnosis is in doubt or detailed mapping of the injury is needed [2]. Here, we report how a combination of US and MRI was used to confirm the diagnosis and guide timely surgical repair. We also compare this case to similar reports and review the importance of prompt intervention in such patients.

## Case presentation

A 55-year-old man with a past medical history of type 2 diabetes mellitus and a myocardial infarction (four years prior, treated with one stent) presented to the emergency department with acute penile pain and swelling. He was not on any anticoagulation or antiplatelet therapy. The patient reported that the injury occurred during sexual intercourse with his wife. During vigorous coitus, he felt a sudden painful snap in his penis when it struck his partner’s perineum, accompanied by immediate pain and bruising. The patient reported a persistent partial erection for 2 hours post-injury before gradual detumescence. On examination, the penis was deviated to the right with ecchymosis and swelling localized to the left lateral shaft. There was no blood at the urethral meatus, and the patient voided spontaneously without difficulty. Given the clinical suspicion for PF, a urinalysis was obtained and showed no hematuria, making urethral injury less likely.

**Figure 1:**
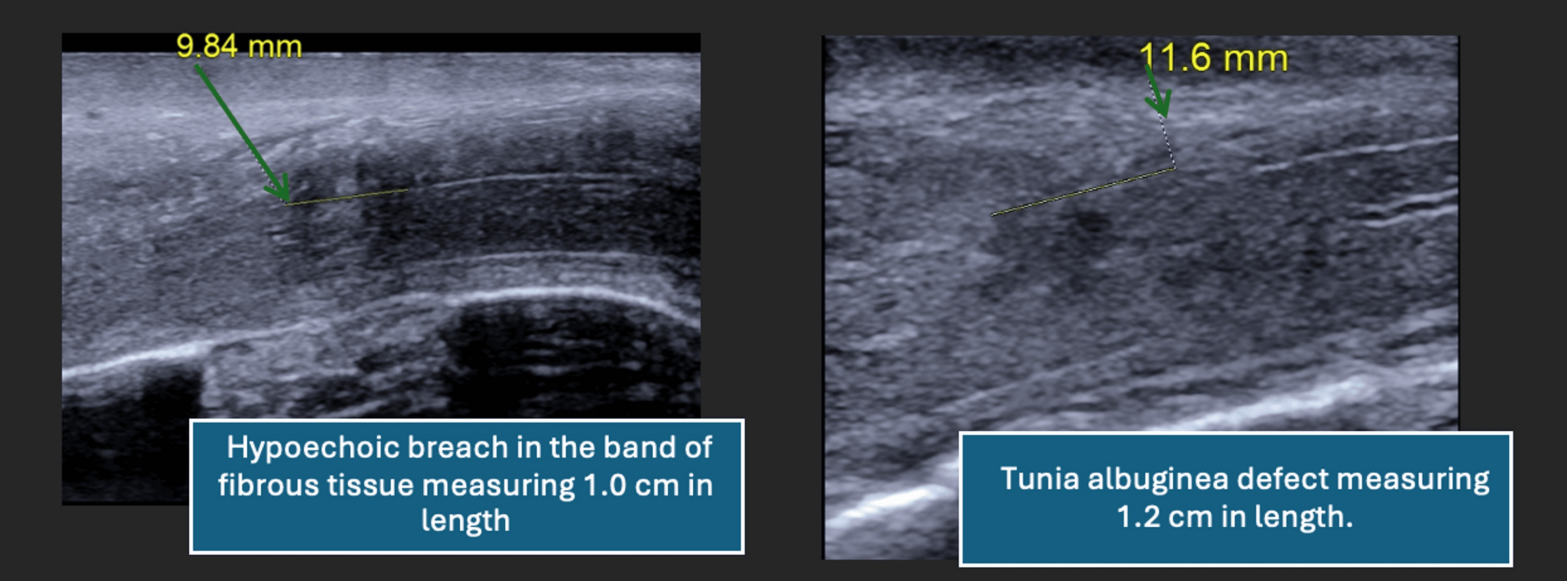
Ultrasound images of tunica albuginea tear. Longitudinal ultrasound views of the left corpus cavernosum demonstrate a hypoechoic defect measuring approximately 1.0 cm (left panel) and 1.2 cm (right panel), consistent with a tunical breach. No hematoma is visualized. The hyperechoic surrounding tissue suggests edema. Arrows denote the site of the tunica albuginea disruption.

A penile US was performed, revealing a hypoechoic defect in the tunica albuginea of the left corpus cavernosum measuring approximately 1.0 cm, with surrounding mild hyperechogenicity suggesting edema (see Figure 1, arrows indicating tear location). No organized hematoma was visualized, and the study was interpreted as equivocal, prompting further workup with an MRI. 

**Figure 2:**
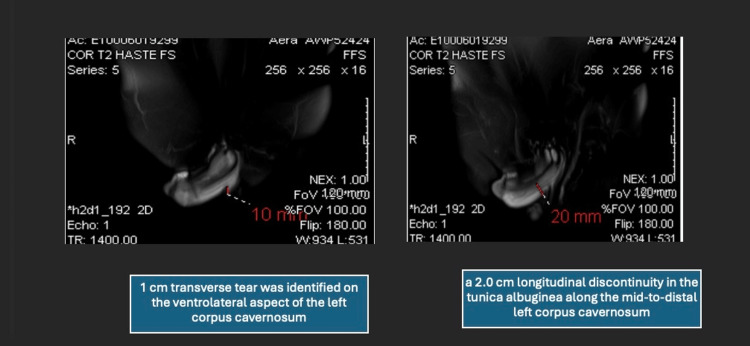
T2-weighted MRI of penile fracture. Axial and sagittal T2-weighted MRI images reveal a 2.0 cm longitudinal discontinuity (right panel) in the tunica albuginea of the mid-to-distal left corpus cavernosum. A contained hematoma measuring 2.0 cm is seen overlying the defect, confined by Buck’s fascia. Urethra is mildly compressed but intact. No contralateral involvement is observed. MRI, magnetic resonance imaging.

An urgent pelvic MRI with contrast was obtained to clarify the extent of the injury (Figure 2, red arrows). MRI demonstrated a 2.0 cm longitudinal discontinuity in the tunica albuginea along the mid-to-distal left corpus cavernosum. Overlying the defect was a 2.0 cm contained hematoma confined by Buck’s fascia, mildly compressing the penile urethra but without evidence of spongiosal or urethral injury. The contralateral corpus cavernosum and midline septum were intact. These imaging findings confirmed an acute PF and enabled precise localization of the defect, facilitating targeted surgical intervention. 

The patient was taken to the operating room within 8 hours of presentation. Through a circumferential subcornal degloving incision, the penis was surgically explored. A 1 cm transverse tear was identified on the ventrolateral aspect of the left corpus cavernosum, corresponding exactly to the MRI findings. No additional corporal or urethral injuries were observed. The tunica albuginea was repaired using interrupted 3.0 absorbable sutures, and the hematoma was evacuated. Postoperatively, the patient was managed conservatively without a Foley catheter and discharged within 24 hours on oral antibiotics and activity restrictions. At follow-up two weeks later, the incision was healing well with improving ecchymosis and no signs of infection or curvature. At three months, the patient reported resumption of normal erectile function, including nocturnal and spontaneous erections, and had resumed sexual activity without difficulty. The physical examination showed no palpable plaques or deformities. He completed the International Index of Erectile Function (IIEF-5) with a score of 24/25, indicating normal erectile function [7].

## Discussion

This case highlights a rare but clinically significant presentation of PF in a middle-aged man. Although PF is a well-documented urologic emergency, its incidence remains low, and cases involving older adults with comorbidities such as type 2 diabetes mellitus and cardiovascular disease are less frequently described in the literature. The classic signs, such as a sudden audible cracking sound, rapid detumescence, and penile swelling, were present in this case, supporting a clinical diagnosis [1]. However, the presence of only partial detumescence and an equivocal US necessitated advanced imaging for confirmation [6].

MRI played a critical role in this case by providing high-resolution visualization of the tunica albuginea tear and associated hematoma. While US remains the first-line imaging modality due to its accessibility and cost-effectiveness, its sensitivity can be limited by factors such as patient habitus, edema, and hematoma [6]. MRI should be considered when sonographic findings are inconclusive or when precise localization of the defect is required for surgical planning [2].

Early surgical intervention within 8 hours of injury has been consistently associated with better functional outcomes and reduced risk of long-term complications such as penile curvature, erectile dysfunction, and painful intercourse [1]. The patient’s favorable recovery aligns with the literature, showing that repairs done within 8 hours lead to significantly lower rates of complications such as erectile dysfunction (reported in 3-7% of early-repair cases) and penile curvature (up to 10%) [1,8]. Average tear sizes in PF typically range from 1.5 to 2.2 cm [8]. Recovery timelines vary, with most patients resuming normal function within 4-6 weeks [8]. Outcomes in older patients or those with comorbidities (e.g., diabetes, cardiovascular disease) are generally less favorable, but early intervention mitigates risk [7,8]. Guidelines from the American Urological Association (AUA) and European Association of Urology (EAU) recommend prompt surgical intervention, imaging only when necessary, and the use of absorbable sutures for tunical repair [7]. Our approach aligned with these protocols. Our patient experienced full recovery of erectile function and reported no deformity or recurrence at follow-up, underscoring the importance of prompt recognition and operative management. This case reinforces the utility of MRI in ambiguous presentations of penile trauma and the importance of a structured approach to diagnosis and treatment. Awareness of atypical features and prompt surgical referral remain key to optimizing outcomes in this rare but impactful urologic emergency.

## Conclusions

PF, while rare, requires a high index of suspicion and prompt surgical intervention to ensure optimal outcomes. This case illustrates the value of MRI in cases where clinical and US findings are inconclusive. Early diagnosis, appropriate imaging, and timely operative management are critical in preventing long-term complications such as erectile dysfunction and penile deformity. Multimodal imaging can enhance diagnostic precision and should be considered when the presentation is atypical.
